# Annotations

**Published:** 1908-08-15

**Authors:** 


### Annotations.

### The Mortality amongst Medical Men.

It is a familiar phrase that he who is his own lawyer has a fool for a client, though doubtless this does not apply to the members of the legal profession themselves. In medicine the corresponding adage is even more true, for it applies to all, doctors included; and therefore the cheap sneer sometimes heard when a medical man is ill or dies is the more misplaced. The physician, in fact, is too wise to attempt his own healing, but presents himself as a patient to some colleague, to whom the problem is quite unaffected by the fact that his patient is one of his own profession. Now it must be admitted that medical men, the preachers of hygiene, ought to be the strictest in their observance of the rules of preventive medicine; and it might be expected that they would in consequence be unusually good "lives," actuarially speaking. It seems, however, that this is not the case, according to the very full and carefully compiled report by the superintendent of statistics, Dr. Tatham, on the subject of occupational mortality in England and Wales during 1900, 1901, and 1902. For although our "comparative mortality figure" is but 952 (the general standard for occupied male workers being 1,000), we die much earlier than, for example, clergymen, lawyers, and farmers. The causes of this high death-rate must be widespread and deep-rooted indeed to overshadow thus the hypothetical advantage of close acquaintance with the laws of health. We may presume they include overwork, irregular hours of work and of meals; insufficient remuneration, and consequent financial worry; exposure to infection whilst below par from fatigue or fasting; insufficient holidays, owing to the objection of patients to deputies. In fact, just those adverse conditions whose avoidance we so often urge on patients.

### Vaccination in New Zealand.

The annual report of the Chief Health Officer for New Zealand for the year 1907, shows clearly that the present attitude of that colony towards vaccination is deplorable in the extreme. We have already drawn attention more than once to the dangerous facilities which now exist in this country for the avoidance of compulsory child-vaccination by the ignorant and misguided parents of the lower classes, and we have indicated the methods by which this state of affairs has been brought about, and who is responsible for it. But the report issued by Dr. J. M. Mason points to a wholesale evasion of vaccination in New Zealand, beside which the condition of things in the United Kingdom seems almost satisfactory. We fear, however, that there is no real cause for self-congratulation over here. A little reflection compels us to suppose that the Colony has merely gone farther along the same dangerous road which the people of the Mother-country are treading, and that unless drastic changes are instituted, we shall soon have to record an equally serious position in England. Last year, out of 24,321 children born in New Zealand, only 4,486 were vaccinated; 2,964 were officially exempted; while 16,871 were "unaccounted for" in this respect—that is to say, they were neither vaccinated nor exempted. Thus over 81 per cent. of the children born in that Colony during 1906-7 are unprotected against small-pox. Whilst in England the President of the Local Government Board has by his recent order rendered exemption the easiest thing in the world to obtain, in New Zealand the great majority of the parents are permitted to evade the law altogether, without even going through the prescribed formality of "conscientious objection." In each country, it is to be observed, vaccination is still styled in official circles "compulsory"; and throughout the empire universal vaccination is still officially held to be the only safeguard against small-pox. It would be difficult to imagine a more gruesome farce. Dr. Mason's commentary on the position in New Zealand is only too pertinent—"To prosecute 69 per cent. of the parents is impracticable; not to carry out the law is demoralising to everyone."

### The Bishops' Appeal.

At the conclusion of the great Pan-Anglican Conference at Lambeth the results of the Bishops' deliberations have been published in a series of 78 resolutions and in the Primate's Encyclical letter. Resolutions 37 to 43, which deal with the more important problems of modern marriage, will have been read far and wide with particular interest. Upon the question of the artificial restriction of the family the Bishops have not hesitated to express their opinion in clear and unmistakable terms. The Conference regards this growing practice with alarm and denounces it as unchristian, immoral, and hostile to the national welfare: "Deliberate tampering with nascent life is repugnant to Christian morality." Here the Conference pays a tribute, which will not fail to be appreciated, to those medical men who have testified against this injurious practice, and appeals to the profession as a whole to co-operate in creating and maintaining a wholesome public opinion on behalf of the reverent use of the married state. There is a time for silence and a time for plain speaking, and the Bishops in council have chosen the course which seemed right to them, and have adopted it without fear. For this they have earned the respect of the medical profession. The weight of medical opinion, even if expressed only here and there has always been upon the side of national efficiency, with which this question is intimately associated; but, as is recognised by the wording of the Bishops' resolutions, the time has come for something more than a passive or semi-passive attitude on the part of clergy and doctors alike, and the two professions must henceforward unite to combat a great and increasing evil. The Pan-Anglican Conference appeals to our profession for active co-operation and support. It realises that, individually and collectively, we wield more influence in this matter than any other body of men, and that it depends upon us whether the resolutions shall become the starting-point of a national reform, or shall remain merely a despairing protest.

